# Supplementary material for: Emergence of the cortical encoding of phonetic features in the first year of life
Source: Nat Commun. 2023 Dec 1;14:7789. doi: 10.1038/s41467-023-43490-x (PMC10692113; doi:10.1038/s41467-023-43490-x)
Supplement: Supplementary file 1 — Supplementary Information [file 41467_2023_43490_MOESM1_ESM.pdf]

Supplementary information

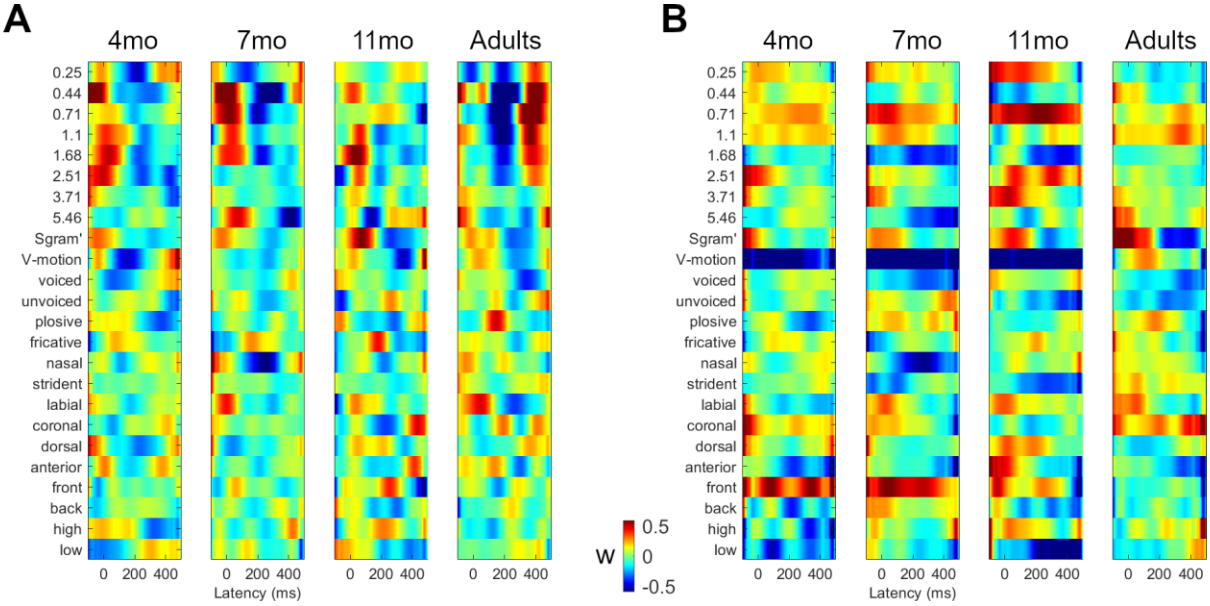

***Supplementary Figure 1**: TRF weights for the acoustic-phonetic model including all features, averaged across all channels, **(A)** in the Δ- and Θ-band (1-8 Hz) and **(B)** in the 0.1-8 Hz band. Each panel reports the average model weights for the n=50 infant participants and the n=17 adult participants. Colours indicate the value of the regression weights, reported on a symmetrical colour-scale from blue (negative weight) to red (positive weight).*

A

S-model

	**lowΔ-band**	**Δ-band**	**Θ-band**
**4mo**	p<0.001, r=0.63, CI=[0.05,0.11]	p<0.001, r=0.63, CI=[0.03,0.05]	p=0.004, r=0.43, CI=[0.001,0.003]
**7mo**	p=0.002, r=0.47, CI=[0.01,0.04]	p=0.007, r=0.41, CI=[0.003,0.02]	p<0.001, r=0.58, CI=[0.001,0.003]
**11mo**	p=0.011, r=0.38, CI=[0.01,0.04]	p=0.011, r=0.38, CI=[0.003,0.02]	p=0.054, r=0.28, CI=[-1.8e-5,0.003]
**Adults**	p=0.017, r=0.36, CI=[0.01,0.04]	p=0.003, r=0.46, CI=[0.01,0.05]	p=0.003, r=0.45, CI=[0.001,0.01]

F-model

	**lowΔ-band**	**Δ-band**	**Θ-band**
**4mo**	p<0.001, r=0.62, CI=[0.02,0.05]	p=0.001, r=0.53, CI=[0.01,0.02]	p=0.170, r=0.20, CI=[-0.3e-4,0.002]
**7mo**	p<0.001, r=0.59, CI=[0.02,0.06]	p<0.001, r=0.67, CI=[0.02,0.03]	p<0.001, r=0.67, CI=[0.001,0.003]
**11mo**	p<0.001, r=0.78, CI=[0.02, 0.04]	p<0.001, r=0.77, CI=[0.02,0.04]	p<0.001, r=0.63, CI=[0.002,0.004]
**Adults**	p=0.027, r=0.33, CI=[0.003,0.05]	p=0.001, r=0.52, CI=[0.02,0.05]	p=0.001, r=0.49, CI=[0.004,0.01]

B

S-model

	**lowΔ-band**	**Δ-band**	**Θ-band**
**4 vs. 11mo**	p=0.024, r=-0.40, CI=[-0.09,-0.02]	p=0.025, r=-0.38, CI=[-0.04,-0.01]	p=0.788, r=-0.06, CI=[-0.002,0.001]
**4 vs. 7mo**	p=0.011, r=-0.47, CI=[-0.08,-0.02]	p=0.007, r=-0.52, CI=[-0.04,-0.01]	p=0.721, r=0.08, CI=[-0.001,0.002]
**7 vs. 11mo**	p=0.89, r=0.03, CI=[-0.02,0.03]	p=0.881, r=0.04, CI=[-0.01,0.02]	p=0.472, r=-0.14, CI=[-0.002,07.1e-4]

F-model

	**lowΔ-band**	**Δ-band**	**Θ-band**
**4 vs. 11mo**	p=0.933, r=-0.01, CI=[-0.02,0.02]	p=0.011, r=0.44, CI=[0.01,0.02]	p=0.011, r=0.45, CI=[8.5e-4,0.004]
**4 vs. 7mo**	p=0.393, r=0.17, CI=[-0.01,0.03]	p=0.195, r=0.25, CI=[-0.002,0.02]	p=0.077, r=0.32, CI=[1.7e-4,0.003]
**7 vs. 11mo**	p=0.335, r=-0.19, CI=[-0.03,0.01]	p=0.45, r=0.15, CI=[-0.01,0.02]	p=0.355, r=0.19, CI=[-5.1e-4,0.003]

Supplementary Table 1*: Table with the post hoc statistical significance results corresponding to the effect sizes in Figure 1B.* ***(A)*** *A post hoc one-sample two-tailed Wilcoxon rank sum test was run to assess if the EEG prediction correlations for each model, group, and frequency-band were greater than zero. The table reports the p-values of that test after FDR-correction, the effect-size of each test, and the confidence interval before FDR-correction. Note that all results were above chance.* ***(B)*** *EEG prediction correlations were compared between groups for each model and frequency-band. The tables report the p-values of two-tailed Wilcoxon rank sum tests after FDR-correction.*
