# Supplementary material for: Emergence of the cortical encoding of phonetic features in the first year of life
Source: Nat Commun. 2023 Dec 1;14:7789. doi: 10.1038/s41467-023-43490-x (PMC10692113; doi:10.1038/s41467-023-43490-x)
Supplement: Supplementary file 3 — Reporting Summary [file 41467_2023_43490_MOESM3_ESM.pdf]

nature portfolio

Corresponding author(s): Giovanni Di Liberto

Last updated by author(s): Sep 13, 2023

Reporting Summary

Nature Portfolio wishes to improve the reproducibility of the work that we publish. This form provides structure for consistency and transparency in reporting. For further information on Nature Portfolio policies, see our Editorial Policies and the Editorial Policy Checklist.

Statistics

For all statistical analyses, confirm that the following items are present in the figure legend, table legend, main text, or Methods section.

n/a	Confirmed	
☐	☒	The exact sample size (*n*) for each experimental group/condition, given as a discrete number and unit of measurement
☐	☒	A statement on whether measurements were taken from distinct samples or whether the same sample was measured repeatedly
☐	☒	The statistical test(s) used AND whether they are one- or two-sided<br>*Only common tests should be described solely by name; describe more complex techniques in the Methods section.*
☐	☒	A description of all covariates tested
☐	☒	A description of any assumptions or corrections, such as tests of normality and adjustment for multiple comparisons
☐	☒	A full description of the statistical parameters including central tendency (e.g. means) or other basic estimates (e.g. regression coefficient) AND variation (e.g. standard deviation) or associated estimates of uncertainty (e.g. confidence intervals)
☐	☒	For null hypothesis testing, the test statistic (e.g. *F*, *t*, *r*) with confidence intervals, effect sizes, degrees of freedom and *P* value noted<br>*Give P values as exact values whenever suitable.*
☒	☐	For Bayesian analysis, information on the choice of priors and Markov chain Monte Carlo settings
☒	☐	For hierarchical and complex designs, identification of the appropriate level for tests and full reporting of outcomes
☐	☒	Estimates of effect sizes (e.g. Cohen's *d*, Pearson's *r*), indicating how they were calculated

Our web collection on statistics for biologists contains articles on many of the points above.

Software and code

Policy information about availability of computer code

Data collection	Experiments were conducted with MATLAB 2017a and the freely available toolkit Psychtoolbox 3.0.14
Data analysis	Analyses were conducted with MATLAB 2021a by using custom scripts developed starting from publicly available scripts shared as part of the CNSP workshop 2021 (Cognition and Natural Sensory Processing; https://cnspworkshop.net). See the code availability statements for the link to the full code

For manuscripts utilizing custom algorithms or software that are central to the research but not yet described in published literature, software must be made available to editors and reviewers. We strongly encourage code deposition in a community repository (e.g. GitHub). See the Nature Portfolio guidelines for submitting code & software for further information.

Data

Policy information about availability of data

All manuscripts must include a data availability statement. This statement should provide the following information, where applicable:

- Accession codes, unique identifiers, or web links for publicly available datasets
- A description of any restrictions on data availability
- For clinical datasets or third party data, please ensure that the statement adheres to our policy

Data was converted to the CND data structure (Continuous-event Neural Data - https://cnspworkshop.net), allowing to carry out the analyses with the CNSP analysis scripts, which provided a platform for bringing together all the necessary libraries. The final data included in the manuscript figures and statistics have been

deposited in the OSF repository https://osf.io/mdnwg. The raw data are not downloadable as they must be considered in conjunction with the data collection videos. As the participants are infants, these video data are confidential data that we do not have ethical permission to make available. Study data were collected and managed using REDCap (Research Electronic Data Capture) electronic data capture tools hosted at Cambridge university.

Research involving human participants, their data, or biological material

Policy information about studies with human participants or human data. See also policy information about sex, gender (identity/presentation), and sexual orientation and race, ethnicity and racism.

Reporting on sex and gender	The experiments involved infant participants and adult participants. We did not expect an effect of sex on phonetic-feature TRFs in the first year of life. Furthermore, previous work with this analysis (phonetic-feature TRF) on adults did not show an effect of sex.
Reporting on race, ethnicity, or other socially relevant groupings	The study did not include a race/ethnicity dimension nor any other socially relevant grouping.
Population characteristics	The present study carried out a re-analysis of an EEG dataset involving a speech listening task in a longitudinal cohort of fifty infants (first part of a larger cohort of 122 subjects21). Participants were infants born full term (37-42 gestational weeks) and had no diagnosed developmental disorder. The experiment involved three EEG recording sessions when the infants (24 male and 26 female) were 4 months old (4mo; 115.6 ± 5.3 days), 7 months old (7mo; 212.5 ± 7.2 days) and 11 months old (11mo; 333.0 ± 5.5 days) [mean ± standard deviation (SD)]. Note that this was a longitudinal investigation, meaning that the same 50 infants were tested at 4, 7, and 11 months of age. In addition to the 150 EEG sessions from the infant dataset, this study also analysed EEG data from twenty-two monolingual, English-speaking adult participants performing the same listening task (11 male, aged 18-30, mean age: 21).
Recruitment	Participants were recruited from a medium sized city in the United Kingdom and surrounding areas via multiple means (e.g., flyers in hospitals, schools, and antenatal classes, research presentations at maternity classes, online advertising), minimising the risk for a self-selection bias.
Ethics oversight	The study,including experiments on adults and infants, was approved by the Psychology Research Ethics Committee of the University of Cambridge

Note that full information on the approval of the study protocol must also be provided in the manuscript.

Field-specific reporting

Please select the one below that is the best fit for your research. If you are not sure, read the appropriate sections before making your selection.

☒ Life sciences ☐ Behavioural & social sciences ☐ Ecological, evolutionary & environmental sciences

For a reference copy of the document with all sections, see nature.com/documents/nr-reporting-summary-flat.pdf

Life sciences study design

All studies must disclose on these points even when the disclosure is negative.

Sample size	Given the many factors of novelty in this study (e.g., first time measuring nursery rhyme TRFs, first time measuring phonetic feature TRFs in infants, first longitudinal mTRF investigation), previous results could not be considered for a formal power analysis. The sample size was set to be the largest as possible within the time-frame of the project. Indeed, that 172 EEG sessions used in the present investigation (N=50 infants, N=22 adults) was a larger sample size than in most previous studies using similar methods (i.e., usually around 10-25 participants).
Data exclusions	Data from five adult participant was excluded due to inconsistencies with the synchronisation triggers, leaving seventeen participants data for the analysis. Data from three infant participants were excluded due to excessive EEG noise.
Replication	This analysis was conducted on the first part of a large cohort of participants. Analyses were conducted with cross-validation within this cohort. Replicability of the results will be determined on the remaining participants at a later stage.
Randomization	This study involved one infant cohort that was studied longitudinally and one cohort of adult participants. No randomisation was required in this case as the study measured healthy development of auditory processing longitudinally, and it did not involve interventions, therapies, or drugs.
Blinding	The study did not involve interventions, therapies, or drugs. So, no blinding was required.

Reporting for specific materials, systems and methods

We require information from authors about some types of materials, experimental systems and methods used in many studies. Here, indicate whether each material, system or method listed is relevant to your study. If you are not sure if a list item applies to your research, read the appropriate section before selecting a response.

Materials & experimental systems

n/a	Involved in the study
☒	☐ Antibodies
☒	☐ Eukaryotic cell lines
☒	☐ Palaeontology and archaeology
☒	☐ Animals and other organisms
☒	☐ Clinical data
☒	☐ Dual use research of concern
☒	☐ Plants

Methods

n/a	Involved in the study
☒	☐ ChIP-seq
☒	☐ Flow cytometry
☒	☐ MRI-based neuroimaging
